# Selected abstracts from the 2021 simulation summit: Virtual. 04–05 November 2021

**DOI:** 10.1186/s41077-021-00189-7

**Published:** 2021-11-01

## 1 Artificial intelligence tutoring versus expert instruction in surgical simulation training: randomized controlled trial

### A. Fazlollahi^1^, R. Del Maestro^1^, J. Harley^1^, A. Alsayegh^1^, M. Bakhaidar^1^, R. Yilmaz^1^, A. Winkler-Schwartz^1^, I. Langleben^1^, N. Mirchi^2^, N. Ledwos^1^

#### ^1^McGill University, Montreal, QC; ^2^University of Toronto, Toronto, ON

##### **Correspondence:** A. Fazlollahi

**Background**

Performance-based assessment in surgical apprenticeship is inefficient and vulnerable to subjectivity. Utilizing Artificial Intelligence (AI) to classify surgical psychomotor expertise, we developed an AI-powered tutor known as the Virtual Operative Assistant (VOA). Being the first intelligent tutoring system in surgical simulation training, VOA’s effectiveness is unknown.

**Objectives**

Determine how learning with the VOA compares with training by remote expert instruction in performing virtual reality brain tumor resections and experiencing emotions and cognitive load.

**Methods**

A multi-institutional randomized controlled trial compared VOA’s automated audiovisual metric-based feedback to remote verbal debriefing with expert instruction and no-feedback controls. Medical students performed six simulated subpial brain tumor resections: five practice attempts followed with feedback and one complex realistic attempt that evaluated skill retention and transfer. A deep learning model, Intelligent Continuous Expertise Monitoring System (ICEMS Expertise Score), and blinded Objective Structured Assessment of Technical Skills (OSATS) evaluated performance. Participants reported emotions before, during and after training and completed a cognitive load questionnaire following training.

**Results**

Seventy medical students from four institutions were randomly assigned to VOA (*n* = 23), Instructor (*n* = 24), and Control (*n* = 23) Groups. 350 practice attempts were assessed by ICEMS, and 70 realistic attempts were evaluated by ICEMS and OSATS. During practice, VOA training resulted in a significant improvement of participants’ Expertise Scores that was on average 0.66 (95% CI 0.55–0.77) and 0.65 (95% CI 0.54–0.77) points higher than Instructor and Control Groups (*p* < .001). Realistic attempt’s average Expertise Score was significantly higher in the VOA group compared to Instructor and Control Groups (mean difference 0.53; 0.49, respectively, *p* < .001). VOA and Instructor Group’s realistic attempt OSATS ratings were not significantly different. OSATS ratings demonstrated that VOA feedback resulted in significantly higher Respect for Tissue and Economy of Movement compared to Control while expert instruction statistically improved Instrument Handling compared to Control. There was no significant between-groups difference in cognitive load, positive-, and negative-activating emotions.

**Conclusion**

VOA’s quantitative automated benchmark feedback demonstrated superior performance outcome, improved skill transfer, with equivalent OSATS ratings and similar cognitive and affective responses compared to remote expert instruction.

## 2 Online simulation is cost effective in training of medical undergraduates

### S. Farahmand^1^, A. Meneghetti^1^, K. Shi^1^, G. Pachev^1^, J. Ramezani^1^, S. Zeinoddini^1^, V. Mehrnoush^1^, S. Hosseinzadeh^1^, H. Kapur^1^, K. Qayumi^1^

#### ^1^University of British Columbia, Vancouver, BC

##### **Correspondence:** S. Farahmand

**Background**

For teaching history-taking skills to novice learners, different educational methods have been used. Standardized Patient (SP) has been considered as a gold standard in medical education. We hypothesized that the use of CyberPatient™ (CP) as an online simulation platform, is as effective as SP.

**Methods**

This study was a randomized controlled trial and the educational effectiveness of CP was compared to SP in improving history taking skills of novice learners. At University of British Columbia (UBC), twenty-two incoming students were randomly divided into two (SP and CP) groups. SP Group (*n* = 11) practiced their history taking skills using the standardized patients and CP Group (*n* = 11) used CyberPatients. For both groups, the content was similar and it included 3 cases of GI pathology with 60 min of study time. The assessment method before and after interventions, included stations of Objective Structured Clinical Examination (OSCE). Data were analyzed in a two way between/within ANOVA and Wald test was used to deal with the violation of the ANOVA assumptions. Economic benefits were assessed as Cost-effectiveness (calculated as Cost/Effect Ratio) and Cost-Value Proposition (Cost-Vale Relationship).

**Results**

Results of this study revealed that in the knowledge domain of history taking, both groups had significant (SP group *p* = 0.006 and CP group *p* = 0.0001) improvement. The history taking knowledge variable in both groups manifested a significant main effect of time indicating that students did better after interventions, F (1,15.1) = 10.5, *p* = 0.011. The groups performed at a similar level after intervention. Moreover, results demonstrated that the use of the CP is more cost-effective and has a better cost/value proposition in medical education.

**Conclusion**

We conclude that CyberPatient™ is as effective as using standardized patients in experiential learning for novice medical students, and it is more cost effective.

## 3 Resuscitation simulation among people who are likely to witness opioid overdose: experiences from the SOONER trial

### J. Whittall^1^, D. Campbell^1^, M. Charles^2^, C. Handford^1^, M. Klaiman^1^, P. Leece^3^, L. Morrison^2^, A. Orkin^1^, C. Strike^1^, S. Turner^3^, A. Wright^3^

#### ^1^University of Toronto, Toronto, ON; ^2^Unity health Toronto, Toronto, ON; ^3^Public health Ontario, Toronto, ON

##### **Correspondence:** J. Whittall

**Background**

The opioid crisis is a growing public health emergency and increasing resources are being directed towards overdose education. Simulation has emerged as a novel strategy for training overdose response, yet little is known about training non-clinicians in bystander resuscitation. Understanding the perspectives of individuals who are likely to experience or witness opioid overdose is critical to ensure that emergency response is effective.

The Surviving Opioid Overdose with Naloxone Education and Resuscitation (SOONER) evaluates the effectiveness of a novel naloxone distribution tool among people who are non-clinicians and likely to witness opioid overdose. Participants’ resuscitation skills are evaluated using a realistic overdose simulation as the primary outcome of the trial.

**Objectives**

The purpose of our study was to understand the experience of participants with the simulation process in the SOONER study.

**Methods**

Our study employed a semi-structured debriefing interview and a follow up qualitative interview to assess the simulation protocol used in the SOONER study and to understand the experience of participants. Interviews were recorded and data pertaining to the experience of participants with the simulation process were extracted. Braun and Clarke’s analytic framework was used to guide a qualitative content analysis which described the experience of participants with the simulation process.

**Results**

21 participants completed the SOONER simulation protocol and 16 of these participants (76%) participated in the follow up qualitative interview, a sample size which achieved thematic saturation. Our qualitative analysis identified 5 themes and 17 subthemes which described the experience of participants within the simulation process. These themes included realism, valuing practical experience, improving self-efficacy, gaining new perspective and bidirectional learning.

**Conclusion**

Our study found that simulation was a positive and empowering experience for participants in the SOONER trial. Our study supports the notion that expanding simulation-based education to non-clinicians may offer an acceptable and effective way of supplementing current opioid overdose education strategies. Increasing the accessibility of simulation-based education may represent a paradigm shift whereby simulation is transformed from a primarily academic practice into a patient-based community resource.

## 5 HERD – Hotwash emergency resuscitation debriefing process at the pediatric emergency department of the Hospital for sick children in Toronto

### M. Lirette^1^, M. Browning^1^, A. Cooney^1^, J. Fayyaz^2^

#### ^1^The Hospital for Sick Children, Toronto, ON; ^2^Toronto, ON

##### **Correspondence:** M. Lirette

**Background**

Immediate critical events debriefing, recommended by international resuscitation guidelines, improves patient outcomes by identifying performance gaps. Standardized debriefing processes through continuous quality improvement methodology helps increase frequency and quality of debriefing.

**Objective**

To implement a structured debriefing process in 60% of eligible events in the emergency department (ED) at The Hospital for Sick Children over a 6-month period to ultimately identify and track performance gaps.

**Methods**

HERD process is being implemented through quality improvement methodology at HSC-ED. Measures include debrief participants and duration (process), number of events debriefed and performance gaps identified (outcome) and user satisfaction (balancing). As part of the HERD process, senior nurses and physicians, with minimal debriefing expertise, are expected to co-lead an immediate short debrief by using a structured debriefing tool called the ‘ED-Hotwash’. This was adapted from an internally developed tool and focuses on identifying individual, team and system-based performance gaps, as recommended by the AHA. ED providers are expected to debrief any resuscitations leading to PICU/NICU admission, Trauma, death or if a team member felt it was warranted. Identified performance gaps are classified based a modified SEIPPS framework and reviewed at monthly meetings where issues are acted upon.

**Results**

There were 22 eligible events that occurred between January and March 2021, including 10 (45%) resuscitations with PICU admission and 9 (41%) trauma activation and most events occurring overnight or on weekends (17/22, 77%). 17/22 events were debriefed (77%) and reasons not to debrief included a “busy ED” or “near handover time”. Most debriefs were led by senior nurses (12/17, 71%) and lasted 15 min on average. 4.8 performance gaps were identified per debrief with most being team or systems based. Equipment usability not being optimized was a common theme across all events. Providers were satisfied with the tool (9.3/10 on 10-point Likert scale) and felt it was “Easy and quick to use” and a “great tool to keep us on track”.

**Conclusion**

Debriefing acute critical events in the ED should be standard of care and implementing a standardized process through quality improvement methodology helps identify and track performance gaps.

## 6 Review of standardized patient scenarios as an effective teaching strategy for implicit bias topics in medical education

### H. Sarvas^1^

#### ^1^Health sciences north, Sudbury, ON

**Background**

Implicit bias is defined as assumptions or actions taken on the basis of stimuli we have repeatedly experienced, causing us to make positive or negative associations about certain groups of people. Implicit bias cannot be eliminated; however, increased awareness of this bias can result in lessened negative outcomes. Implicit bias training during medical education is an optimal time as learners appreciate this knowledge as foundational to their practice. As medical schools enhance curriculum surrounding implicit bias one suggestion includes integrating structured clinical examinations with standardized patients embracing implicit bias topics.

Research Question:

Are standardized patients case scenarios an effective teaching strategy for teaching implicit bias to medical learners?

**Methods**

A search was conducted in databases PubMed, CINAHL, and Medline. Dates were limited to articles published between 2011 and 2021. Inclusion criteria was: published in English, peer-reviewed, medical students as the majority learner population, standardized patient scenarios as the primary teaching strategy, and addressing the topic of implicit bias. Initially, 56 articles were collected; three were removed as duplicates. 45 were excluded via abstract or full-text review based on inclusion criteria set above. Eight papers were included in the final analysis.

**Results**

Implicit bias populations addressed varied including: The Lesbian Gay Bisexual Transgender+ population, patients with low health literacy, Black persons, persons living with HIV/AIDS, and persons who are obese. All studies used pre- and post-encounter surveys following standardized patient encounters to assess efficacy of teaching strategy. The majority of studies noted a significant improvement in empathy or attitudes surrounding the client population after undergoing the education. Four studies noted improved comfort, knowledge and/or skills in working with the patient population after the education. Common limitations included lack of: longitudinal analysis to assess long-term effects, control group or baseline for intervention, validated tools, and generalizability.

**Conclusion**

Standardized patients have the potential to be part of the implicit bias curriculum, among other teaching strategies. Exact implicit bias topics to be addressed need further evaluation. It is uncertain whether the integration of implicit bias education via standardized patient case scenarios impacts long-term behavior change, or patient outcomes for medical learners, and warrants further investigation.

## 7 Using rapid cycle deliberate practice simulation to teach basic life support in pediatric intensive care: a pilot randomized control trial

### N. Oomen^1^, N. Pereira^2^, J. Duff^2^

#### ^1^Stollery Children’s hospital, Sherwood Park, AB; ^2^Stollery Children’s hospital, Edmonton, AB

##### **Correspondence:** N. Oomen

**Background**

Chest compressions are a critical intervention for patients in a cardiac arrest state. The quality of compressions provided during a resuscitation is a crucial predictor of neurologically-intact survival. In 2015, members of the Stollery Children’s Hospital Pediatric Intensive Care Unit (PICU) participated in a multicenter study looking at chest compression quality (Cheng, Brown et al. 2015). The results of this study showed poor compression performance by PICU staff when compared to the Heart and Stroke Foundation of Canada’s (HSFC) Cardiopulmonary Resuscitation (CPR) guidelines.

**Objectives**

To improve the chest compression quality of Stollery PICU Staff, specifically in relation to compression depth, a key metric associated with CPR survival.

**Design**

This study was conducted as a prospective, randomized control trial.

**Intervention**

The intervention group received simulation based Basic Life Support (BLS) training using the feedback technique known as, Rapid Cycle Deliberate Practice (RCDP). The control group also received simulation based BLS training but with traditional post simulation feedback. Both groups received training with simulation scenarios specific to a pediatric critical care environment. CPR quality in each group was measured pre- and post-course using a Laerdalä QCPR Mannequin. Data analyzed using 2-way mixed methods ANOVA.

**Results**

There were 41 participants in the RCDP group (72.5% RNs, 17.5% RRTs, 10% other) and 41 in the control group (76% RNs, 17% RTs, 7% other). Data was missing from 6 participants in the control arm due to a technical error. Pre-course percentage of compressions with adequate depth (50-60 mm) was 73.3% and 53.1% in the control and intervention group respectively. This increased to 80.5% (control) and 78.1% (intervention) post-course. Both groups improved significantly (*p* < 0.01) with more improvement noted in the RCDP arm (group*time interaction *p* < 0.05). For chest compression rate, both groups improved significantly (68.2% to 79.6% control and 63.7% to 78.5% RCDP; *p* < 0.05) but there was no difference between the groups.

**Conclusion**

A simulation-based RCDP BLS course resulted in improved CPR performance immediately post-course compared to a course using traditional simulation debriefing techniques.

## 9 Entrustable professional activities (EPAs) for simulation faculty?! A novel approach to standardizing mentorship and faculty development for healthcare simulation programs

### A. Kaba^1^, C. Serieska^1^, T. Cronin^1^, J. Semaka^1^, C. Eichorst^2^, T. Fuselli^1^, N. Terpstra^3^, M. Johnson^4^, V. Grant^1^

#### ^1^Alberta health services, Calgary, AB; ^2^Alberta health services, Edmonton, AB; ^3^Alberta health services, Red Deer, AB; ^4^Alberta health services, grand prairie, AB

##### **Correspondence:** A. Kaba

**Background**

The simulation-based education (SBE) literature mostly emphasizes debriefing frameworks and methods, with little discussion detailing how simulation-based education competencies develop over time. Despite its importance, simulation faculty development concentrates primarily on foundational skills, such as debriefing, and neglects to describe trajectory through which simulation faculty develop these skills from novice to independent practice.

Introduced in 2005, by the Royal College of Physicians and Surgeons of Canada, as part of competency-based medical education (CBME), Entrustable Professional Activities (EPAs) offer a robust curriculum development and assessment process for workplace-based assessments. While there is emerging of evidence on the development and application of EPAs for medical residents, there is paucity of literature on EPAs specifically for faculty across their healthcare simulation career. In alignment with CBME, eSIM provincial program addressed this gap by developing a novel set of EPAs, and milestones specifically targeting competencies for SBE.

**Objective**

Objective of this curricular innovation project was to use a modified Delphi technique to develop valid and reliable EPAs and milestones that a simulation faculty is trusted to independently perform by the end of faculty development mentorship program.

**Methods**

Using a modified Delphi technique, the team identified 14 expert simulation faculty across the province to rate the level importance of each of the EPAs and milestones. Validating EPAs for SBE is important to ensure both the accuracy of the observation but also the reliability of the task as observed by rater in performing the assessment.

**Results**

Five EPAs were identified as part of a trajectory through faculty development: Technology; Scenario Design; Setting the Stage and Prep (including In situ, Lab Considerations and Learner Readiness); Prebriefing and Debriefing. The EPAs provide a structured framework of clear expectations for assessing and tracking achievements of simulation faculty; targeting areas for improvement and formative feedback to facilitate independent and safe practice.

**Conclusion**

While mapping of EPAs and milestones have been traditionally used for residency training, this novel curricular development of EPAs for simulation faculty training, illustrates an education innovation to advance standard competencies in SBE. The tool provides opportunities for significant advancements in transforming faculty development for simulation programs.

## 10 In-situ simulation and rapid-cycle testing for assessment of a code gray: Covid-19 vaccine freezer failure policy to prevent loss of vaccine supply

### S. DeSousa^1^, V. May^2^, A. Ryzynski^2^, D. Vella^2^, C. Wong^2^

#### ^1^Sunnybrook health sciences Centre, Etobicoke, ON; ^2^Sunnybrook health sciences Centre, Toronto, ON

##### **Correspondence:** S. DeSousa

**Background**

Sunnybrook Health Sciences Centre was a premier site for the storage, distribution and administration of the Pfizer-BioNTech COVID-19 vaccine. Proper storage of the vaccine requires an ultra-low temperature freezer (− 70 °C). The Department of Pharmacy was responsible for vaccine storage and developed a Code Gray: Vaccine Freezer Failure policy; a Code Gray is initiated when a hospital experiences loss or failure of essential services. The Sunnybrook Canadian Simulation Centre (SCSC) led in-situ simulations to help Pharmacy test their novel policy and ability to transfer vaccines quickly and successfully in the case of freezer failure to prevent vaccine supply loss. The policy involved multiple key stakeholders, including Pharmacy Stores Manager, Pharmacy technicians, Pharmacists, Quality & Patient Safety, Risk, Emergency Preparedness, Patient Transport, Telecommunications and Security. The process required numerous steps over a large geographical area to safely relocate vaccine supply.

**Objective**

To assess the usability of an organizational policy for Code Gray: Covid-19 Vaccine freezer failure.

**Methods**

The Model for Improvement (MFI) was the scientific method chosen to guide process improvement for this project. In-situ simulation with rapid-cycle testing was utilized as the assessment method for quality improvement. Wearable cameras, direct observation and debriefing were the methods utilized for data collection.

Simulated Code Grays were initiated and stakeholders activated to test response times, communication, equipment familiarity and ease of policy use. Data was analyzed and presented to the stakeholders for reflection. Areas for change were identified, modifications made and a plan was developed and initiated through several PDSA cycles.

**Results**

Over 22 policy improvement items concerning communication, human factors and equipment were identified. Threats to policy deployment, vaccine access, vaccine transport and equipment were identified. All items were addressed to mitigate the risk of vaccine supply loss.

**Conclusion**

In-situ simulation is effective in testing and identifying latent threats in policy design and deployment. MFI and rapid-cycle testing are essential methods to iteratively test, assess and act to inform healthcare policy development and improvement.

## 11 CIRCumcision (training) using simulation (CIRCUS) - report of a pilot simulation-based platform for newborn circumcision

### A. Alsabban^1^, K. Martin^1^, M. Maizels^2^, M. Chua^1^, M. Pokarowski^1^, T. Greenspoon^1^, J. Knabl^1^, W. Farhat^3^, E. Louca^1^, M. Perlmutar^4^, M. Rickard^1^, A. Lorenzo^1^, E. Smith^5^, A. Varghese^1^, J. dos Santos^1^

#### ^1^The Hospital for Sick Children, Toronto, ON; ^2^Northwestern university Feinberg school of medicine, Chicago, IL; ^3^University of Wisconsin school of medicine and public health, Madison, WI; ^4^Michael Garron hospital, Toronto, ON; ^5^Emory university school of medicine, Atlanta, GA

##### **Correspondence:** A. Alsabban

**Background**

Complications resulting from newborn circumcisions, such as bleeding and penile injury, may be under-reported. Such complications likely occur due to the absence of structured training.

**Objective**

We sought to create a pilot training platform consisting of 1. Online didactic learning; 2. Simulation practice; and 3. Hands-on clinical performance. Herein we present our results of CIRCUS for newborns.

**Methods**

Study design. 1. Knowledge. We created an online learning platform for carrying out newborn circumcisions. We used this online resource to develop a set of 25 pre-test questions. 2. Simulation. 3D silicone replica of a newborn size penis with an intact foreskin model was printed in order to practice Mogen & GOMCO clamps. In order to adapt to COVID restrictions, simulation training was offered either virtually (four participants) or in-person (four participants). 3. Clinical. Upon completion of simulation training, learners performed real-time clinical circumcisions under the direct supervision of experts. Outcome measures included pre−/post- knowledge scores, self-competence questionnaires, and skill assessments of simulation and clinical performances (Likert rating). Face validity for training success was determined by showing an 80% passing score for knowledge and > 75% (mostly independent) performance for both simulation and clinical circumcisions.

**Results**

For this pilot, we restricted enrollment to pediatric residents (7) and a nurse practitioner. Wilcoxon Sum Rank test for non-parametric paired samples for pre and post-knowledge test showed a positive difference for all participants with a median of 20 points increase (*p* = 0.011). Both in-person and virtual participants performed > 75% of the simulation and clinical circumcisions independently. For self-efficacy, Z scores assessment for changes of mean values from baseline and mean difference (changes from baseline) as the effect measure, and the results favor post-CIRCUS except for the management of bleeding.

**Conclusion**

This pilot of CIRCUS learning shows face validity for both in-person and virtual training for newborn circumcision. More We plan to extend this platform to include more trainees and offer it to established practitioners. We believe that the availability of formal training will ultimately reduce adverse outcomes.

